# Prognostic implications of coronary artery disease and stress tests in patients with elevated left ventricular filling pressure and preserved ejection fraction

**DOI:** 10.3389/fcvm.2022.955731

**Published:** 2022-08-15

**Authors:** Jeong Hun Seo, David Hong, Taeho Youn, Seung Hun Lee, Ki Hong Choi, Darae Kim, Taek Kyu Park, Joo Myung Lee, Young Bin Song, Jin-Oh Choi, Joo-Yong Hahn, Seung-Hyuk Choi, Hyeon-Cheol Gwon, Eun-Seok Jeon, Jeong Hoon Yang

**Affiliations:** ^1^Division of Cardiology, Department of Medicine, Samsung Medical Center, Sungkyunkwan University School of Medicine, Seoul, South Korea; ^2^Division of Cardiology, Department of Internal Medicine, Kangwon National University Hospital, Kangwon National University School of Medicine, Chuncheon, South Korea; ^3^Department of Critical Care Medicine, Samsung Medical Center, Sungkyunkwan University School of Medicine, Seoul, South Korea

**Keywords:** coronary artery disease, stress test, heart failure with preserved ejection fraction, myocardial ischemia, mortality

## Abstract

**Background:**

The prognostic role of myocardial ischemia in patients with heart failure with preserved ejection fraction (HFpEF) has not been fully elucidated. Therefore, we investigated the change in echocardiographic parameters and clinical outcomes based on the presence of epicardial coronary artery disease (CAD) and positive stress tests in HFpEF patients.

**Methods:**

Symptomatic patients with left ventricular end diastolic pressure ≥15 mmHg who underwent coronary angiography were analyzed between January 2000 and August 2019 after exclusion of patients with acute coronary syndrome.

**Results:**

A total of 555 HFpEF patients were invasively confirmed, 285 (51%) had angiographically-proven CAD. HFpEF patients with CAD displayed greater deterioration in left ventricular ejection fraction (*p* = 0.002) over time but this was not observed in those without CAD (*p* = 0.99) on follow-up echocardiography; however, the mitral annulus early diastolic velocity (e') was significantly decreased in both groups (*p* < 0.001 and *p* = 0.003, respectively). Among 274 patients that received stress tests, those with positive stress tests showed a decline in e' (*p* 0.001), but *t*his was not found in subjects with negative stress tests (*p* = 0.44). There was no significant difference in all-cause mortality between patients with CAD and without CAD (*p* = 0.26) with a median follow-up of 10.6 years.

**Conclusion:**

In HFpEF patients, CAD was associated with greater deterioration in the left ventricular systolic function but not with mortality during the follow-up. In addition, myocardial ischemia with a positive stress test may contribute to greater deterioration of diastolic dysfunction.

## Introduction

Heart failure (HF) with preserved ejection fraction (HFpEF) remains a poorly understood clinical syndrome without effective targeted therapies (1). The clinical syndrome of HFpEF develops from a complex interaction of several risk factors such as aging, obesity, hypertension, myocardial ischemia, and arterial stiffness that cause organ dysfunction and, ultimately, clinical symptoms (2). Myocardial ischemia is basically driven by an impaired myocardial oxygen supply-demand balance due to macrovascular (epicardial) coronary artery disease as well as coronary microvascular dysfunction (3, 4). Recently, numerous studies have suggested that coronary microvascular dysfunction may play an important role in the pathophysiology of HFpEF (5–7). Even in HFpEF patients without significant epicardial coronary stenosis, recent studies have showed that a decreased coronary flow reserve is associated with worse diastolic dysfunction and outcomes (8).

Although clinical cardiology originally focused on diseases of the large epicardial conduit arteries, only a few studies have reported on the association between epicardial coronary artery disease (CAD) and clinical outcomes in HFpEF patients, and the results are inconsistent (9, 10). While there were more events in the CAD group than the no-CAD group, the difference was not statistically significant in the previous HFpEF study (10), and another study showed that CAD was associated with increased mortality and greater deterioration in ventricular function, however, patients that received complete revascularization had similar mortality rates compared to those without CAD (9). Furthermore, a positive stress test was interpreted as reflective of microvascular ischemia, although this may have been falsely positive in HFpEF patients without CAD (11–13).

Therefore, we sought to investigate the change in cardiac functions using echocardiographic parameters over time and clinical outcomes based on the presence of CAD and positive stress tests in symptomatic patients with left ventricular end diastolic pressure (LVEDP) ≥15 mmHg who underwent coronary angiography and left cardiac catheterization.

## Methods

### Study population

**Figure 1:**
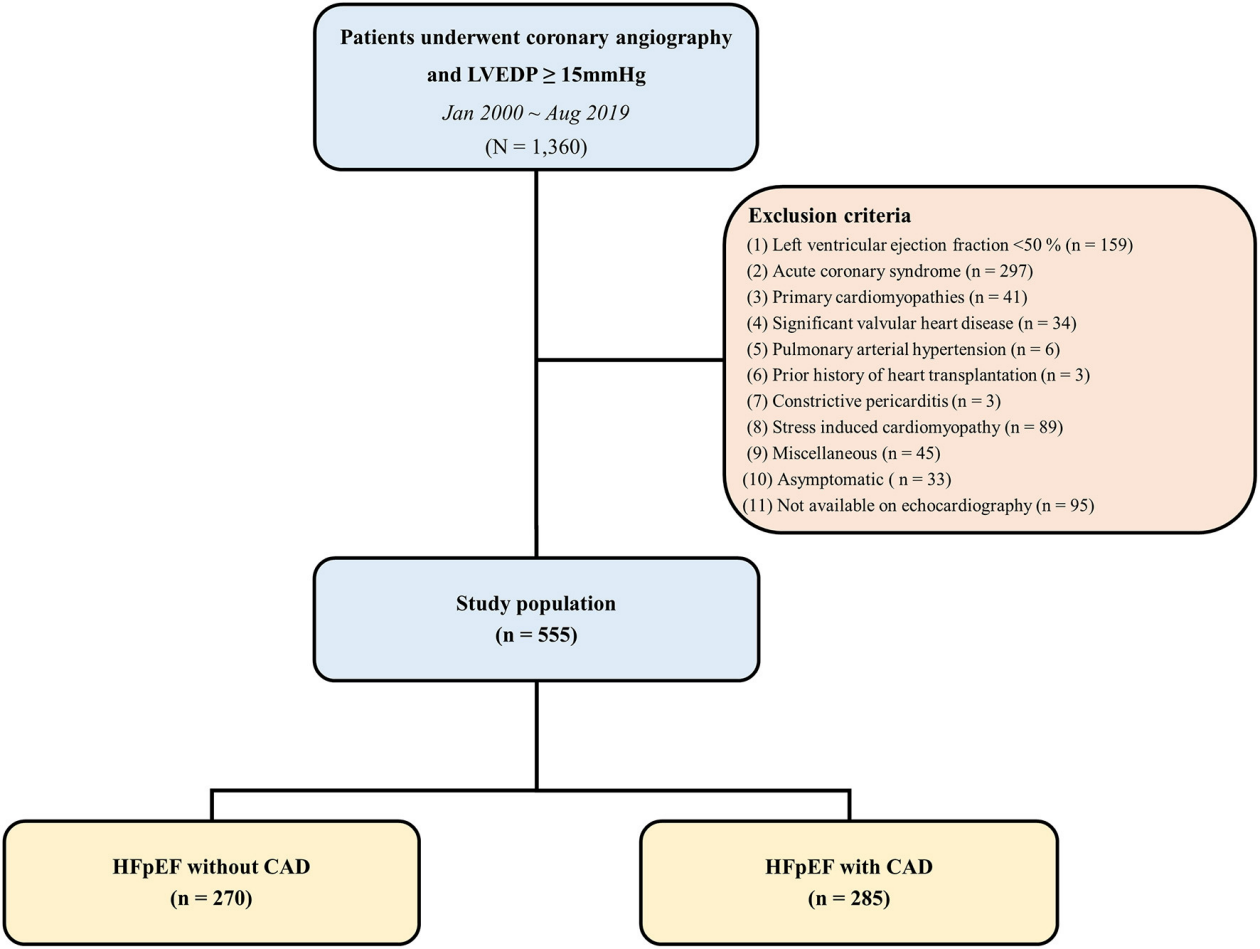
Flow diagram. CAD, coronary artery disease; HFpEF, heart failure with preserved ejection fraction; LVEDP, left ventricular end diastolic pressure.

We identified all patients whose LVEDP was measured in the catheterization laboratory database of a tertiary hospital between January 2000 and August 2019. Among these patients, we screened symptomatic patients with left ventricular ejection fraction (LVEF) ≥50% on pre-angiography transthoracic echocardiography, and elevated diastolic filling pressure (LVEDP ≥15 mmHg) based on cardiac catheterization. The definition of symptomatic patients was the case with dyspnea, fatigue, dizziness, chest discomfort, and ankle edema. Patients with acute coronary syndrome, primary cardiomyopathies such as dilated cardiomyopathy and hypertrophic cardiomyopathy, amyloidosis, significant valvular heart disease (greater than mild stenosis, moderate left-sided regurgitation), pulmonary arterial hypertension, heart transplantation, constrictive pericarditis, stress-induced cardiomyopathy, isolated right-sided heart failure, high output heart failure, myocarditis, ventricular arrhythmia, aortic dissection and asymptomatic patients were excluded. A total of 555 patients were divided into those with and without epicardial CAD, defined by luminal stenosis of >50% on coronary angiography or previous coronary artery revascularization (Figure 1). This study was approved by the institutional ethical review board of Samsung Medical Center (IRB File No. 2019-11-191).

### Data collection and outcomes

The baseline demographic, echocardiographic, laboratory, and follow-up clinical outcome data were collected retrospectively through medical record review. Positive stress tests on non-invasive stress testing was defined as ST-segment depression >1 mm on treadmill test, new regional wall motion abnormalities on echocardiography, or reversible perfusion defects on myocardial nuclear or magnetic resonance imaging. The primary outcome was all-cause mortality. We also assessed the change of systolic and diastolic function using echocardiography based on the presence or absence of CAD, and positive or negative of stress tests.

### Two-dimensional and Doppler echocardiography

Comprehensive transthoracic echocardiography was performed on commercially available equipment (Vivid 7, GE Medical System, Milwaukee, WI or Acuson 512, Siemens Medical Solution, Mountain View, CA or Sonos 5500, Philips Medical System, Andover, MA, USA). Standard M-mode, two-dimensional and color Doppler imaging were performed in parasternal, suprasternal, substernal, and apical views with positional adjustment of the patients. In the case of atrial fibrillation, at least three consecutive beats from three standard apical views were collected for analysis. The mean data of three beats was used for the final analysis. The first and last echocardiograms taken during the study period were used to compare the change of echocardiographic parameters. Anatomic measurements were made according to the American Society of Echocardiography Guidelines. We assessed measurement reproducibility in 20 randomly selected cases by repeated measurements.

### Statistical analysis

Continuous variables were reported as the mean ± standard deviation and were compared using the Welch's *t*-test or the Wilcoxon rank sum test, as appropriate. Categorical variables were summarized by frequencies or percentages and analyzed with the Chi-square test or Fisher's exact test, as appropriate. The changes of echocardiographic parameters were analyzed with the paired *t*-test. The cumulative incidence of clinical events was evaluated by Kaplan-Meier analyses and the level of significance was assessed with the log-rank test. Statistical analyses were performed using R Statistical Software (version 3.5.2; R Foundation for Statistical Computing, Vienna, Austria) and SPSS 22.0 statistical software (SPSS Inc., Chicago, IL, USA) with *p* < 0.05 considered statistically significant.

## Results

### Baseline characteristics

A total of 555 HFpEF patients were invasively confirmed, 285 (51%) had angiographically-proven CAD. Baseline characteristics were presented in Table 1. Compared with HFpEF patients without CAD, those with CAD were more likely to be men, and to be treated with anti-ischemic medicines, including beta-blockers, nitrates, statins, and aspirin. HFpEF patients without CAD had more dyspnea, fatigue, and chest discomfort than those with CAD. There were no significant differences in comorbidities such as diabetes, hypertension, dyslipidemia, and smoking history between HFpEF patients with and without CAD. The laboratory test results indicated that there were no significant differences between the two groups. A total of 274 (49%) patients with HFpEF underwent stress testing before angiography. Among the patients that underwent stress testing, 122 (78%) of those with angiographically-proven CAD were found to have ischemia at the time of stress testing. Conversely, 46 (39%) of HFpEF patients with no significant CAD on angiography were found to have a positive test. LVEDP by left cardiac catheterization was higher in patients with CAD than those without CAD.

**Table 1:** Baseline characteristics.

	**HFpEF with CAD (*n* = 285)**	**HFpEF without CAD (*n* = 270)**	***P*-value**
Male	194 (68)	137 (51)	**<0.001**
Age (yrs)	63 ± 11	62 ± 13	0.237
Body mass index, kg/m^2^	25.5 ± 3.5	25.2 ± 3.6	0.333
Systolic blood pressure, mmHg	137 ± 20	131 ± 22	**0.002**
**Clinical symptoms**			
Dyspnea	96 (34)	127 (47)	**0.001**
NYHA II	57 (20)	64 (24)	
NYHA III	29 (10)	43 (16)	
NYHA IV	10 (3.5)	18 (6.7)	
Fatigue	7 (2.5)	17 (6.3)	**0.026**
Dizziness	20 (7.0)	23 (8.5)	0.509
Chest discomfort	58 (20)	92 (34)	**<0.001**
Ankle edema	17 (6.0)	19 (7.0)	0.608
**Medical history**			
Diabetes	120 (42)	98 (36)	0.198
Hypertension	160 (56)	131 (49)	0.072
Smoking ever	67 (24)	61 (23)	0.381
Chronic kidney disease	19 (6.7)	20 (7.4)	0.733
Dyslipidemia	98 (34)	85 (32)	0.467
Atrial fibrillation	18 (6.3)	33 (12)	**0.038**
**Medications**			
ACE inhibitor	64 (23)	31 (12)	**0.001**
Angiotensin II receptor blocker	93 (33)	82 (30)	0.567
Beta blocker	149 (52)	87 (32)	**<0.001**
Loop diuretics	35 (12)	40 (15)	0.383
Aldosterone antagonist	24 (8.4)	25 (9.3)	0.728
Statin	194 (68)	89 (33)	**<0.001**
Nitrate	125 (44)	30 (11)	**<0.001**
Aspirin	247 (87)	102 (38)	**<0.001**
Anticoagulation	13 (4.6)	18 (6.7)	0.280
**Laboratories**			
Hemoglobin, g/dl	13.4 ± 1.9	13.2 ± 2.0	0.160
Creatinine, g/dl	1.1 ± 0.7	1.2 ± 1.2	0.694
Glucose, mg/dl	123 ± 46	123 ± 49	0.999
Uric acid, mg/dl	5.5 ± 1.7	5.5 ± 1.6	0.940
CRP, mg/dl	0.62 ± 1.52	0.93 ± 2.76	0.142
NTproBNP, pg/ml (*n* = 174/172)	193 (86–800)	182 (62–729)	0.214
Troponin I, ng/ml (*n* = 186/160)	0.032 (0.007–0.150)	0.150 (0.018–0.150)	0.470
**Stress test, total number/positive ischemia**			
Total (*n* = 274)	157/122 (78)	117/46 (39)	**<0.001**
Treadmill test (*n* = 90)	51/37 (73)	39/17 (44)	**0.005**
Echocardiography (*n* = 64)	26/12 (46)	38/14 (37)	0.456
Nuclear (*n* = 102)	79/72 (91)	23/10 (44)	**<0.001**
MRI (*n* = 18)	1/1 (100)	22/5 (29)	0.146
**Invasive hemodynamics**			
LVEDP, mmHg	20.5 ± 4.6	19.5 ± 4.5	**0.009**

Values are presented as mean ± standard deviation or number (%) or median (interquartile range).

ACE, angiotensin converting enzyme; CAD, coronary artery disease; CRP, C-reactive protein; HFpEF, heart failure with preserved ejection fraction; LVEDP, left ventricular end-diastolic pressure; MRI, magnetic resonance imaging; NTproBNP, N-terminal pro-B-type natriuretic peptide.

Statistical significance was defined as P < 0.05 by Welch's t-test (continuous variables) or the Chi-square t-test (categorical variables). Bold formatting of values indicates the presence of statistical significance (P < 0.05).

### Echocardiographic and angiographic assessment

Left ventrucular end diastolic/systolic diameter, late diastolic mitral inflow velocity, and right ventricular systolic pressure were slightly greater in HFpEF patients with CAD compared to those without CAD (Table 2). A total of 148 (51%) patients in the CAD group had multivessel disease. The left anterior descending artery was the most commonly involved vessel. Finally, 230 (81%) of the CAD group received complete revascularization at previous and index procedures and the residual SYNTAX score ≤ 8 was in 262 (92%) patients.

**Table 2:** Echocardiographic and angiographic evaluation.

	**HFpEF with CAD (*n* = 285)**	**HFpEF without CAD (*n* = 270)**	***P*-value**
**Echocardiography**			
Left ventricular end diastolic diameter, mm	50.9 ± 4.8	49.7 ± 4.8	**0.005**
Left ventricular end systolic diameter, mm	31.6 ± 5.3	30.1 ± 4.5	**0.001**
Left ventricular mass index, g/m^2^	106.6 ± 26.5	100.7 ± 26.1	0.089
Left ventricular ejection fraction, %	61.8 ± 7.2	63.0 ± 6.9	**0.049**
Left atrial volume index, ml/m^2^	38.1 ± 16.0	39.5 ± 16.3	0.426
Early diastolic mitral inflow velocity, m/s	0.68 ± 0.21	0.69 ± 0.23	0.435
Late diastolic mitral inflow velocity, m/s	0.80 ± 0.21	0.73 ± 0.21	**<0.001**
Mitral annulus early diastolic velocity, m/s	0.063 ± 0.019	0.066 ± 0.022	0.265
Early diastolic velocity of the mitral annulus ratio	12.1 ± 5.1	11.5 ± 4.8	0.279
Right ventricular systolic pressure, mm Hg	33.9 ± 8.9	31.6 ± 8.7	**0.036**
Tricuspid annular peak systolic velocity, cm/s	13.2 ± 2.6	12.9 ± 2.8	0.411
**Angiography**			
Extent of coronary artery disease			
1-vessel disease	139 (49)		
2-vessel disease	82 (29)		
3-vessel disease	64 (22)		
**Disease territory**			
Left main coronary artery	23 (8.1)		
Left anterior descending artery	195 (68)		
Left circumflex artery	133 (47)		
Right coronary artery	157 (55)		
Complete revascularization	230 (81)		
SYNTAX score	11.9 ± 9.6		
Residual SYNTAX score ≤ 8	262 (92)		

Values are presented as mean ± standard deviation or number (%).

CAD, coronary artery disease; HFpEF, heart failure with preserved ejection fraction.

Statistical significance was defined as P < 0.05 by Welch's t-test (continuous variables) or the Chi-square t-test (categorical variables). Bold formatting of values indicates the presence of statistical significance (P < 0.05).

### Echocardiographic parameter changes

**Figure 2:**
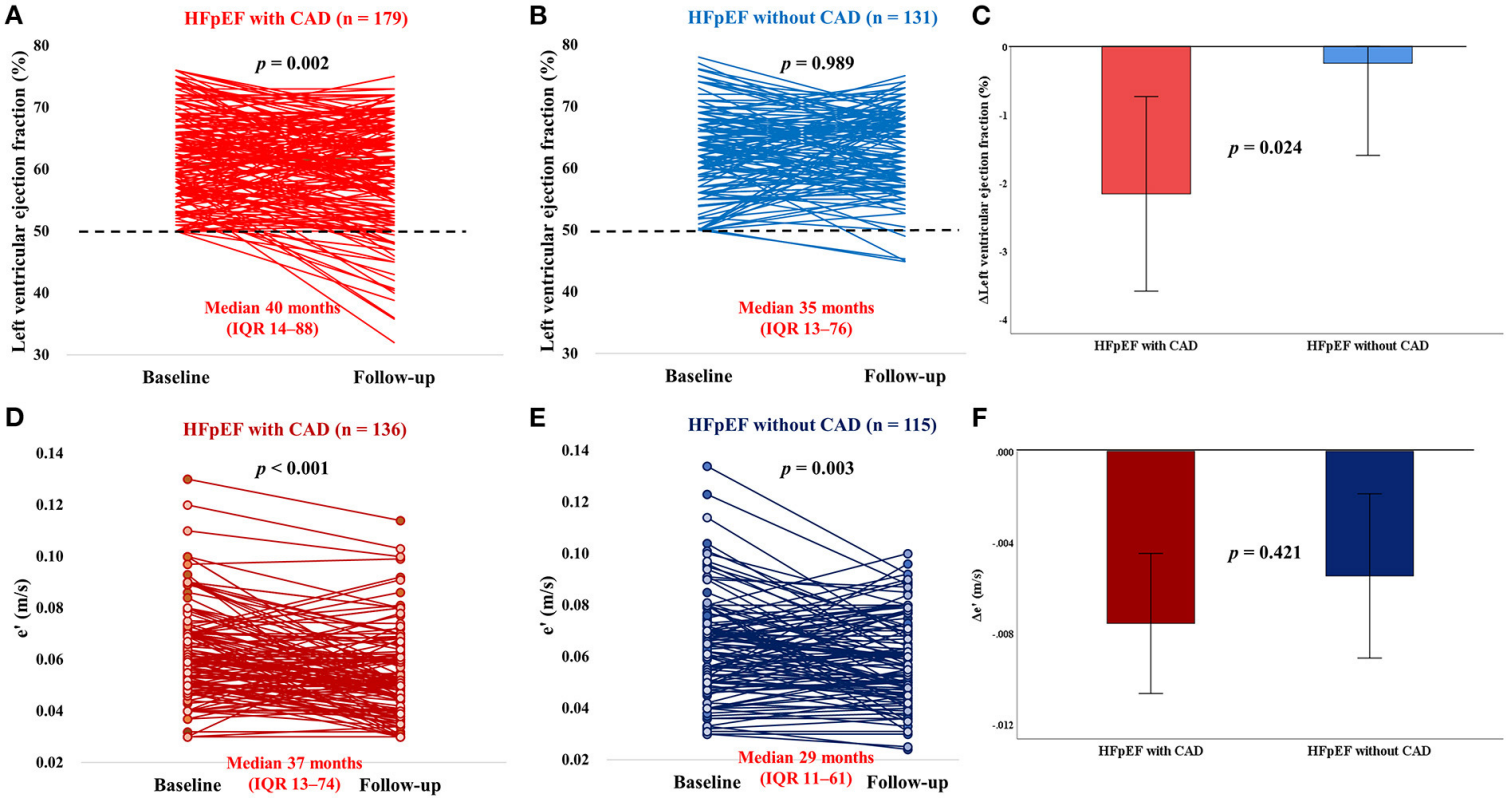
Impact of CAD on longitudinal changes in LVEF and e'. CAD, coronary artery disease; HFpEF, heart failure with preserved ejection fraction; LVEF, left ventricular ejection fraction; e', mitral annulus early diastolic velocity. **(A–C)** In patients with HFpEF without significant CAD, there was no longitudinal change in EF, whereas in patients with CAD, there was a reduction in LVEF, with multiple patients developing reduced LVEF (<50%, dotted lines). **(D–F)** The e' was significantly decreased in both HFpEF patients with CAD and without CAD, and there were no significant diffrences in the decline of e' between the two groups.

**Figure 3:**
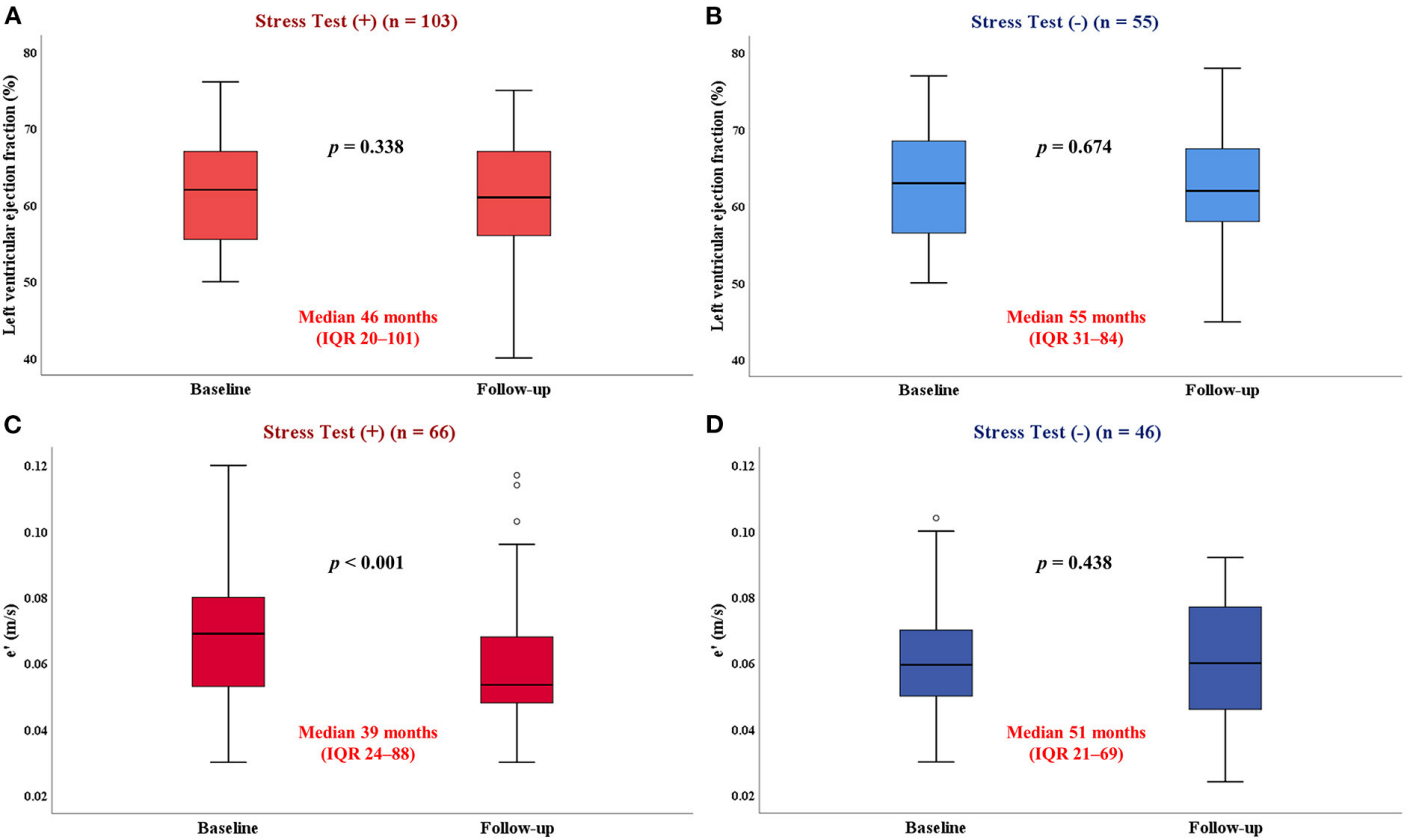
Impact of stress tests on longitudinal changes in LVEF and e'. LVEF, left ventricular ejection fraction; e', mitral annulus early diastolic velocity. **(A,B)** There were no changes in the LVEF according to the results of stress tests. **(C,D)** HFpEF patients with positive stress tests had a significant decrease of the e', while there was no significant decrease in those with negative stress tests.

Repeat echocardiography was performed during a median interval of 37 months (IQR: 12–80 months) after catheterization. The LVEF deteriorated in patients with CAD but not in those without CAD (Figures 2A,B). Compared with patients without CAD, HFpEF patients with CAD experienced a greater decline in LVEF over time (−2.1 ± 8.8% vs. −0.1 ± 8.1%; *p* = 8 0.024) (Figure 2C). The mitral e' velocity, a good indicator of left ventricular relaxation, significantly decreased in both HFpEF patients with CAD and without CAD (*p* < 0.001 and *p* = 0.003, respectively; Figures 2D,E). There were no significant differences in the decline 11 of mitral e' velocity between the two groups (−0.007 ± 0.017 vs. −0.005 ± 0.020; *p* = 0.421) (Figure 2F). There were no changes in the LVEF regardless of positive stress test (Figures 3A,B). However, patients with positive stress tests had a significant decrease of the mitral e' velocity, while there was no significant decrease in those with negative stress tests (*p* 0.001 and *p* = 0.44, respectively; Figures 3C,D). In subgroup analysis, HFpEF patients with positive stress tests and without CAD showed no change in LVEF and a significant decrease in the mitral e' velocity (Supplementary Figure 1). The changes in other echocardiographic parameters according to the presence of CAD or postive stress tests were also presented (Supplementary Figures 2, 3).

### Clinical outcomes

**Figure 4:**
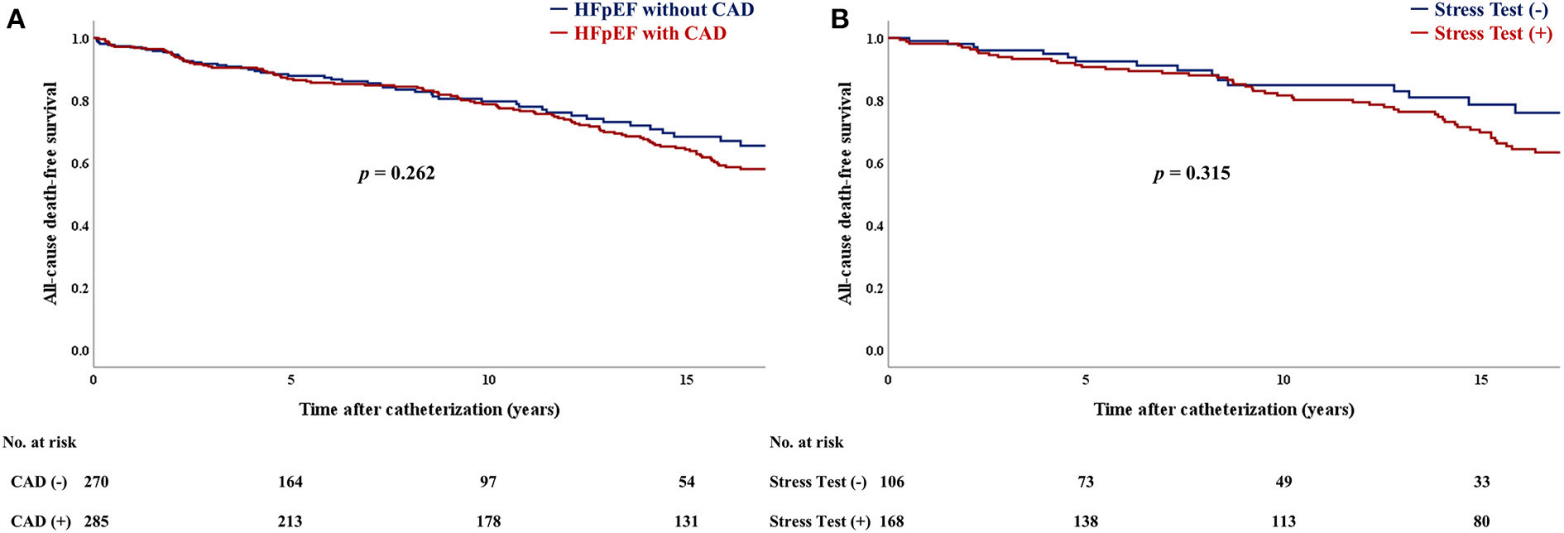
Kaplan–Meier curves for **(A)** all-cause mortality between CAD and no-CAD groups. Kaplan–Meier curves for **(B)** all-cause mortality between stress test (+) and (-) groups. CAD, coronary artery disease; HFpEF, heart failure with preserved ejection fraction.

During a median follow-up of 10.6 years (IQR: 3.7–17.0 years), there were 166 deaths due to any cause. All-cause mortality showed no significant difference between HFpEF patients with CAD and those without CAD (*p* = 0.26; Figure 4A). There was no statistically significant difference in the primary outcome, according to stress testing results (*p* = 0.32; Figure 4B). In addition, there were no significant differences in the composite outcome of cardiovascular death and hospitalization due to HF (Supplementary Figure 4).

### Reproducibility

Measurements of LVEF and mitral e' velocity were repeated in a randomly selected subgroup of 20 patients for analysis of reproducibility. The intraclass correlation coefficients for intra-observer variability of LVEF and mitral e' velocity measurements were 0.975 and 0.988, respectively.

## Discussion

In this study, we investigated the change in cardiac structures and clinical outcomes according to the presence or absence of epicardial CAD and positive or negative stress tests in symptomatic patients with elevated left ventricular filling pressure who underwent coronary angiography and left cardiac catheterization. The major findings of this study were as follows: (1) HFpEF patients with epicardial CAD had a greater deterioration of LVEF over time although this was not significant in those without CAD, while mitral e' velocity was decreased in both groups regardless of the presence of CAD; (2) the mitral e' velocity declined in patients with positive stress tests, but was unchanged in those with negative stress tests; (3) there were no significant differences in long-term mortality according to the presence of epicardial CAD or positive stress tests.

Myocardial ischemia associated with multiple comorbidities such as epicardial CAD, coronary microvascular dysfunction, or other non-cardiac conditions, such as chronic kidney disease, or chronic obstructive pulmonary disease, is recognized as a potential pathophysiological mechanism for developing HFpEF (5). Approximately 50% of patients with symptoms or signs of ischemia referred for elective coronary angiography are found to have non-obstructive CAD (2) and these patients are more prone to hospitalizations as a result of heart failure even in patients with preserved ejection fraction (14). To date, previous studies have primarily investigated the relationship between coronary microvascular dysfunction-induced myocardial ischemia/injury and clinical outcomes, rather than the large conduit of the epicardial artery (4, 8, 15). Traditionally, clinical cardiology focused on the large conduit of epicardial artery to determine a prognosis of various cardiovascular conditions. In the context of HFpEF, CAD is common (16, 17) and is considered an important risk factor for HFpEF patients (9). A report from the Coronary Artery Surgery Study showed that the extent of angiographically-confirmed CAD had a definite prognostic impact in patients with symptoms of HF and LVEF ≥45% at the 6-year follow-up (18). In comparison, HFpEF patients that achieved complete revascularization had lower mortality rates, and outcomes that were not significantly different, compared with those without CAD, although the CAD group experienced significantly worse survival compared with the no-CAD group, with a median follow-up of 4 years (9). Because of conflicting findings on the relationship between CAD and clinical outcomes from previous studies in HFpEF patients, we investigated this relationship in an invasively-confirmed HFpEF cohort. This present study shows that the presence of CAD is not associated with long-term mortality with a median follow-up of around 10 years. One strength of this study is that it presents results for longer follow-up periods in a relatively large number of patients, compared with previous studies. This finding may be attributable to the fact that the majority of patients with CAD received complete revascularization and had a residual SYNTAX score ≤ 8 after coronary revascularization. Furthermore, it was conducted on patients with stable ischemic heart disease, excluding acute coronary syndrome. The International Study of Comparative Health Effectiveness with Medical and Invasive Approaches, which was a recent randomized trial, demonstrated that an initial invasive strategy compared with an initial conservative strategy did not confer a reduced risk for cardiovascular death or myocardial infarction in patients with stable ischemic heart disease (19). Accordingly, findings from previous studies and our study suggest that patients with a significant relief of myocardial ischemia caused by epicardial CAD may have a similar prognosis to those without epicardial CAD even in the context of HFpEF with definitely elevated LV filling pressure.

Over a median follow-up of 37 months, the presence of CAD was associated with greater reduction in LVEF over time, confirming and extending recent studies (9, 20). Progressive atherosclerosis in patients with significant CAD causes anatomical and functional ischemia, which can lead to impaired cardiac systolic and diastolic functions (21). In our study, the presence of CAD has also shown a decline in mitral e' velocity over time. This finding supports the ischemic cascade that coronary hypoperfusion leads to changes in myocardial metabolism, followed by diastolic dysfunction and systolic dysfunction (22). The mitral e' velocity significantly decreased over time, regardless of whether CAD was present, whereas patients with positive stress tests showed a significant decrease in mitral e' velocity over time, but no significant difference was noted for those with negative stress tests. The mitral e' velocity is an indicator that reflects ventricular relaxation and is less affected by factors such as preload, compared with other echocardiographic parameters that reflect left ventricular diastolic function (23). In a study of athletes, those with abnormal stress tests that were indicative for myocardial ischemia without obstructive CAD had a lower coronary flow reserve, which is likely to reflect coronary microvascular dysfunction (24). Indeed, non-obstructive coronary atherosclerosis is observed in up to 50% of patients with angina and positive stress test results that undergo diagnostic coronary angiography (25). In our study, the decline of mitral e' velocity in the positive stress test group supports the hypothesis that myocardial ischemia secondary to coronary microvascular dysfunction may contribute to greater deterioration of diastolic dysfunction in HFpEF (12, 15).

There were some limitations to this study. First, all participants were required to undergo coronary angiography at a single tertiary center, which could induce referral bias, and which may have influenced the prevalence of CAD. Second, this study is not a protocolized study because it is conducted with retrospective analysis, thus, it is difficult to sufficiently correct the selection bias and adjust time dependent variables. In particular, the lack of standardization in follow-up echocardiography time could lead to unfair findings. Accordingly, this study limits the ability to make conclusions on the causal effects of CAD on ventricular function or outcome in HFpEF patients. Therefore, well-designed, prospective studies are required to confirm our findings. Third, the definition of CAD was only based on angiographic appearances, which are well-known to be inaccurate, not by physiologic assessment. Finally, not all patients received follow-up echocardiography and stress testing. Among the total 555 patients, stress test was performed in 274 patients, and follow-up echocardiography were performed in 165 patients who received stress tests. Patients with follow-up echocardiography in those received stress tests seem to be too small to draw conclusive findings. Hence, a large-scale prospective study is needed in the future.

In conclusion, epicardial CAD was associated with greater deterioration in left ventricular systolic function over time, but not long-term mortality in invasively-confirmed HFpEF patients that received optimal anti-ischemic treatments. Furthermore, myocardial ischemia, represented by a positive stress test, may contribute to greater deterioration of diastolic dysfunction.

## Data availability statement

The original contributions presented in the study are included in the article/Supplementary material, further inquiries can be directed to the corresponding author.

## Ethics statement

The studies involving human participants were reviewed and approved by the Institutional Ethical Review Board of Samsung Medical Center (IRB File No. 2019-11-191). Written informed consent for participation was not required for this study in accordance with the national legislation and the institutional requirements.

## Author contributions

All authors contributed to the conception and interpretation of data, drafting of the manuscript, revising it critically for important intellectual content, and final approval of the manuscript.

## Conflict of interest

The authors declare that the research was conducted in the absence of any commercial or financial relationships that could be construed as a potential conflict of interest.

## Publisher's note

All claims expressed in this article are solely those of the authors and do not necessarily represent those of their affiliated organizations, or those of the publisher, the editors and the reviewers. Any product that may be evaluated in this article, or claim that may be made by its manufacturer, is not guaranteed or endorsed by the publisher.

## Supplementary material

Supplementary Figure 1 The changes of **(A)** LVEF and **(B)** e' in HFpEF patients with positive stress tests and without CAD. CAD, coronary artery disease; HFpEF, heart failure with preserved ejection fraction; LVEF, left ventricular ejection fraction; e', mitral annulus early diastolic velocity.

Supplementary Figure 2 The changes in E/e' ratio, LAVI, and RVSP between **(A)** CAD and **(B)** no-CAD groups. CAD, coronary artery disease; E/e', early diastolic velocity of the mitral annulus; LAVI, left atrial volume index; RVSP, right ventricular systolic pressure.

Supplementary Figure 3 The changes in E/e' ratio, LAVI, and RVSP between **(A)** stress test (+) and **(B)** (-) groups. E/e', early diastolic velocity of the mitral annulus; LAVI, left atrial volume index; RVSP, right ventricular systolic pressure.

Supplementary Figure 4 Kaplan–Meier curves for cardiovascular death and hospitalization due to heart failure **(A)** between CAD and no-CAD groups, **(B)** between stress test (+) and (-) groups. CAD, coronary artery disease; HFpEF, heart failure with preserved ejection fraction.

## Abbreviations

CAD: Coronary artery disease
e': Mitral annulus early diastolic velocity
HFpEF: Heart failure with preserved ejection fraction
LVEDP: Left ventricular end diastolic pressure
LVEF: Left ventricular ejection fraction.
