# Supplementary figures and images for: Prognostic implications of coronary artery disease and stress tests in patients with elevated left ventricular filling pressure and preserved ejection fraction
Source: Front Cardiovasc Med. 2022 Aug 15;9:955731. doi: 10.3389/fcvm.2022.955731 (PMC9421048; doi:10.3389/fcvm.2022.955731)

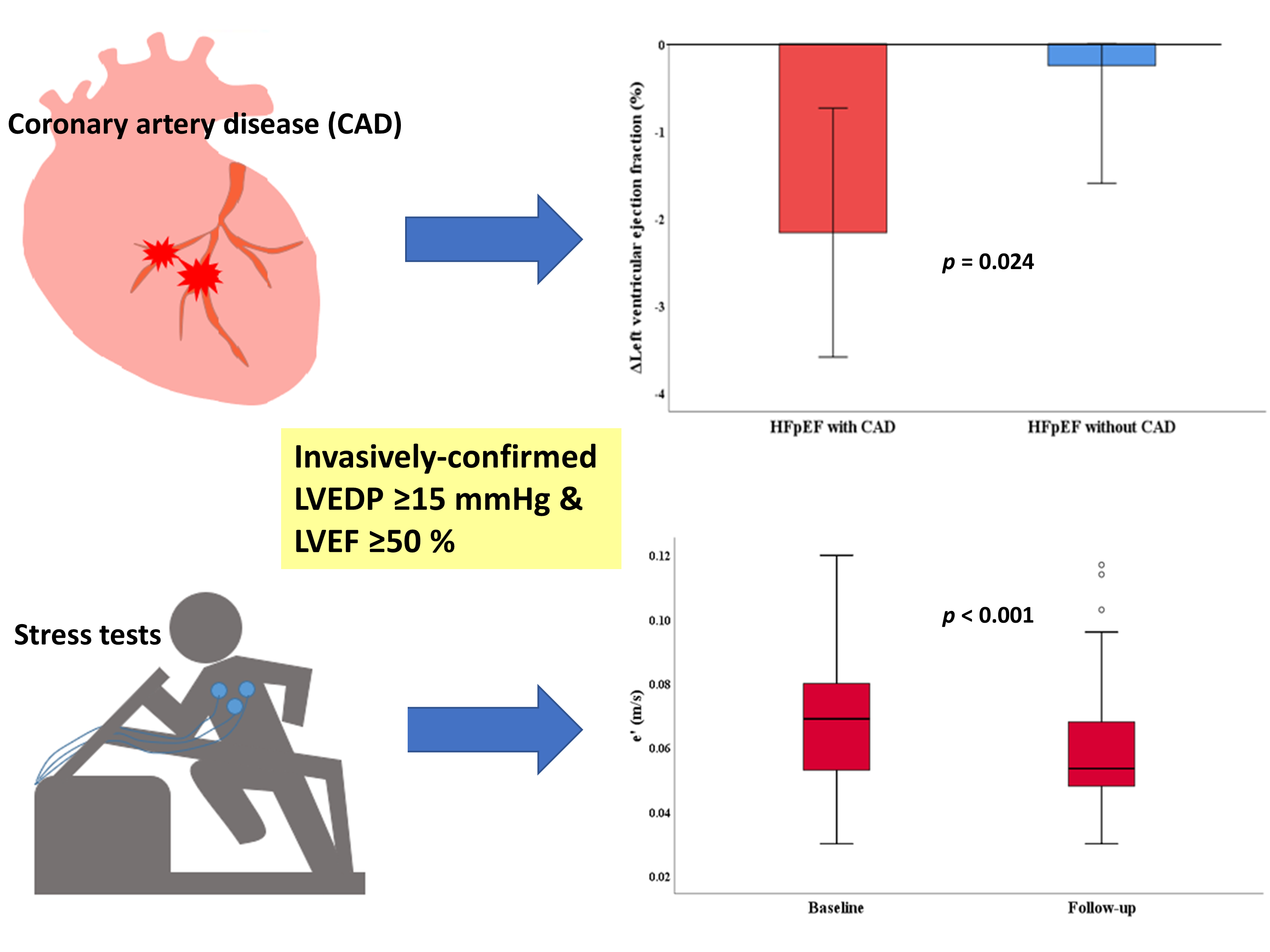

Supplement: Supplementary Figure 1 — The changes of (A) LVEF and (B) e' in HFpEF patients with positive stress tests and without CAD. CAD, coronary artery disease; HFpEF, heart failure with preserved ejection fraction; LVEF, left ventricular ejection fraction; e', mitral annulus early diastolic velocity. [file Image_1.TIF]

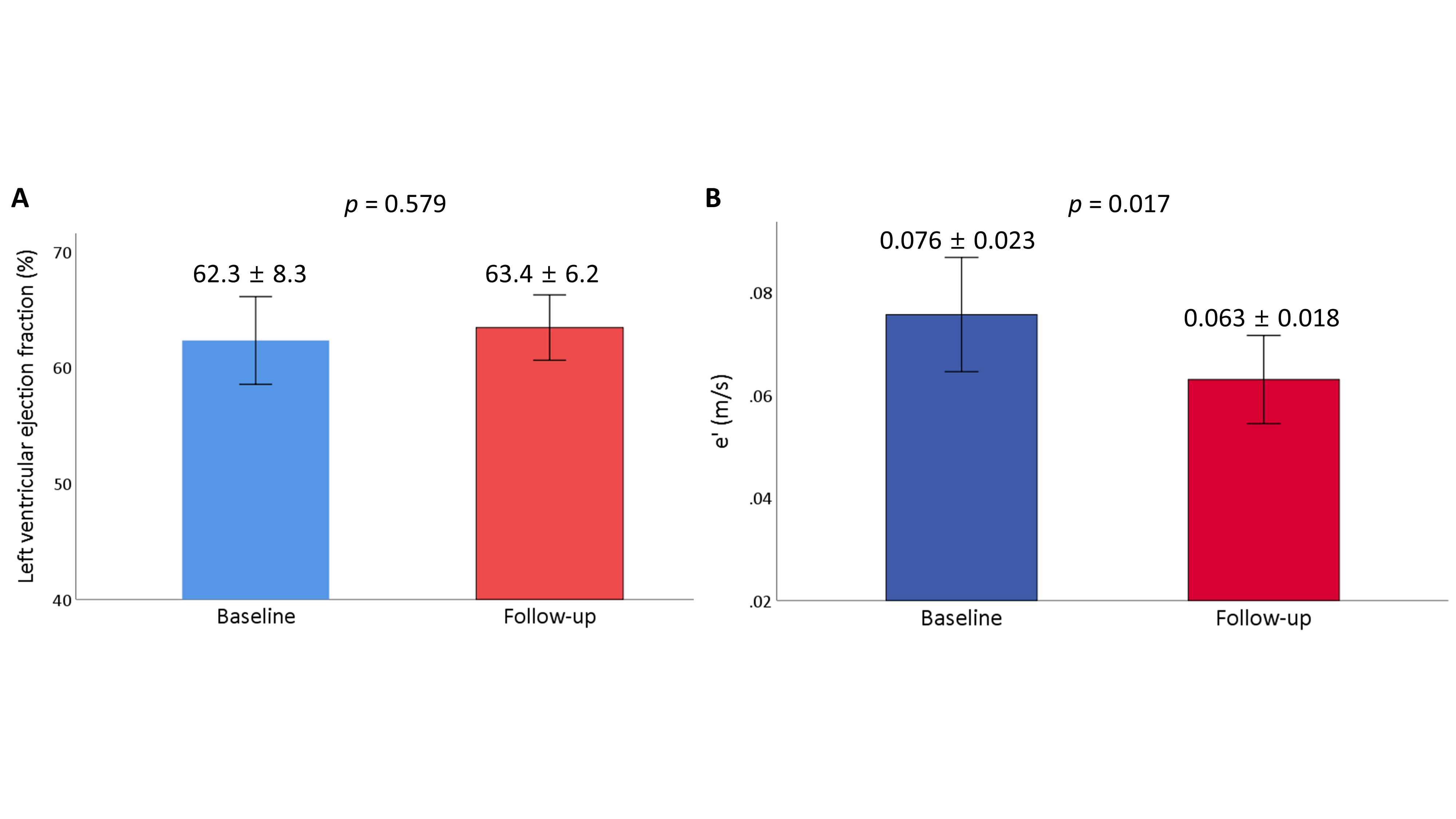

Supplement: Supplementary Figure 2 — The changes in E/e' ratio, LAVI, and RVSP between (A) CAD and (B) no-CAD groups. CAD, coronary artery disease; E/e', early diastolic velocity of the mitral annulus; LAVI, left atrial volume index; RVSP, right ventricular systolic pressure. [file Image_2.TIF]

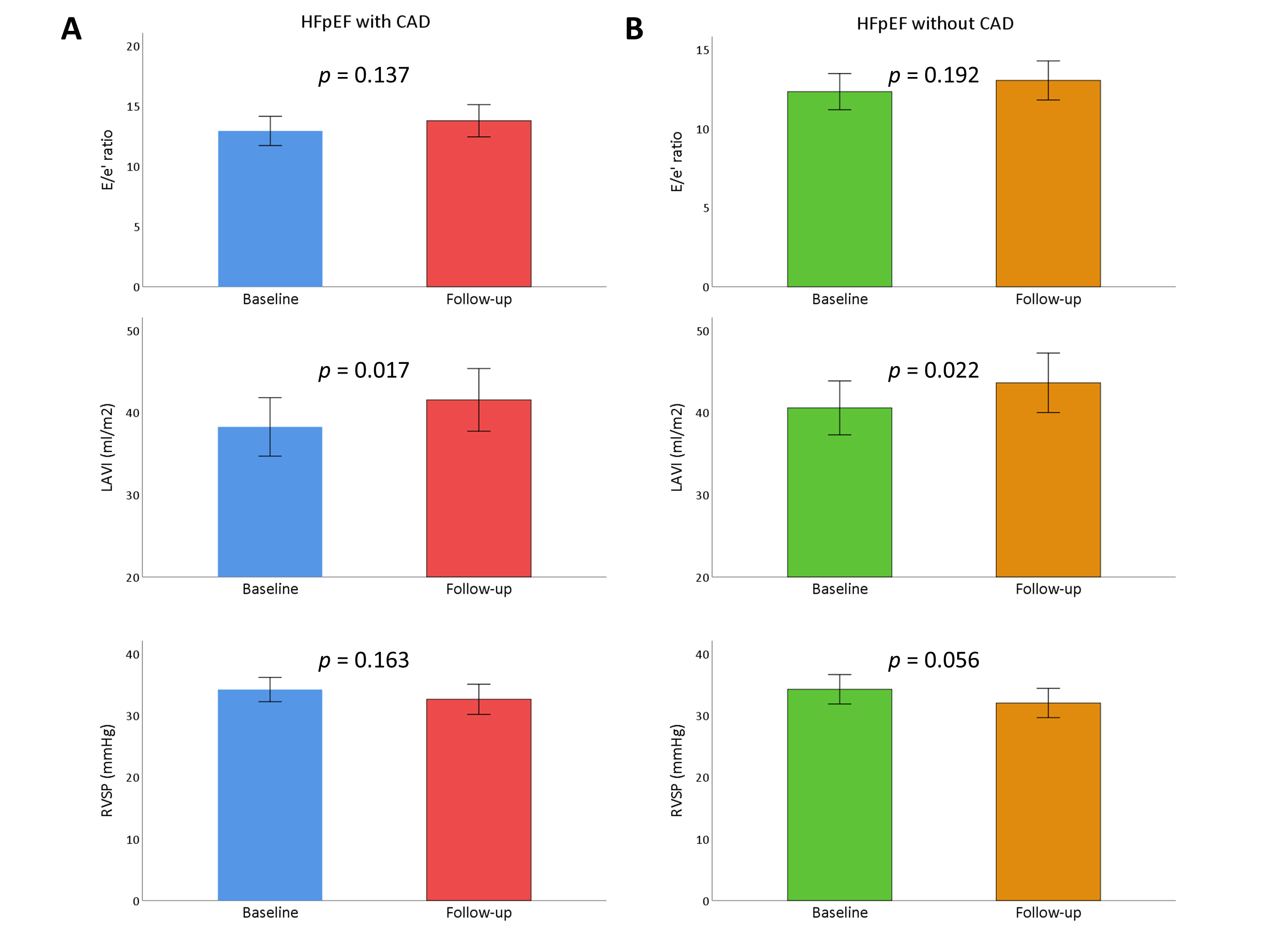

Supplement: Supplementary Figure 3 — The changes in E/e' ratio, LAVI, and RVSP between (A) stress test (+) and (B) (-) groups. E/e', early diastolic velocity of the mitral annulus; LAVI, left atrial volume index; RVSP, right ventricular systolic pressure. [file Image_3.TIF]

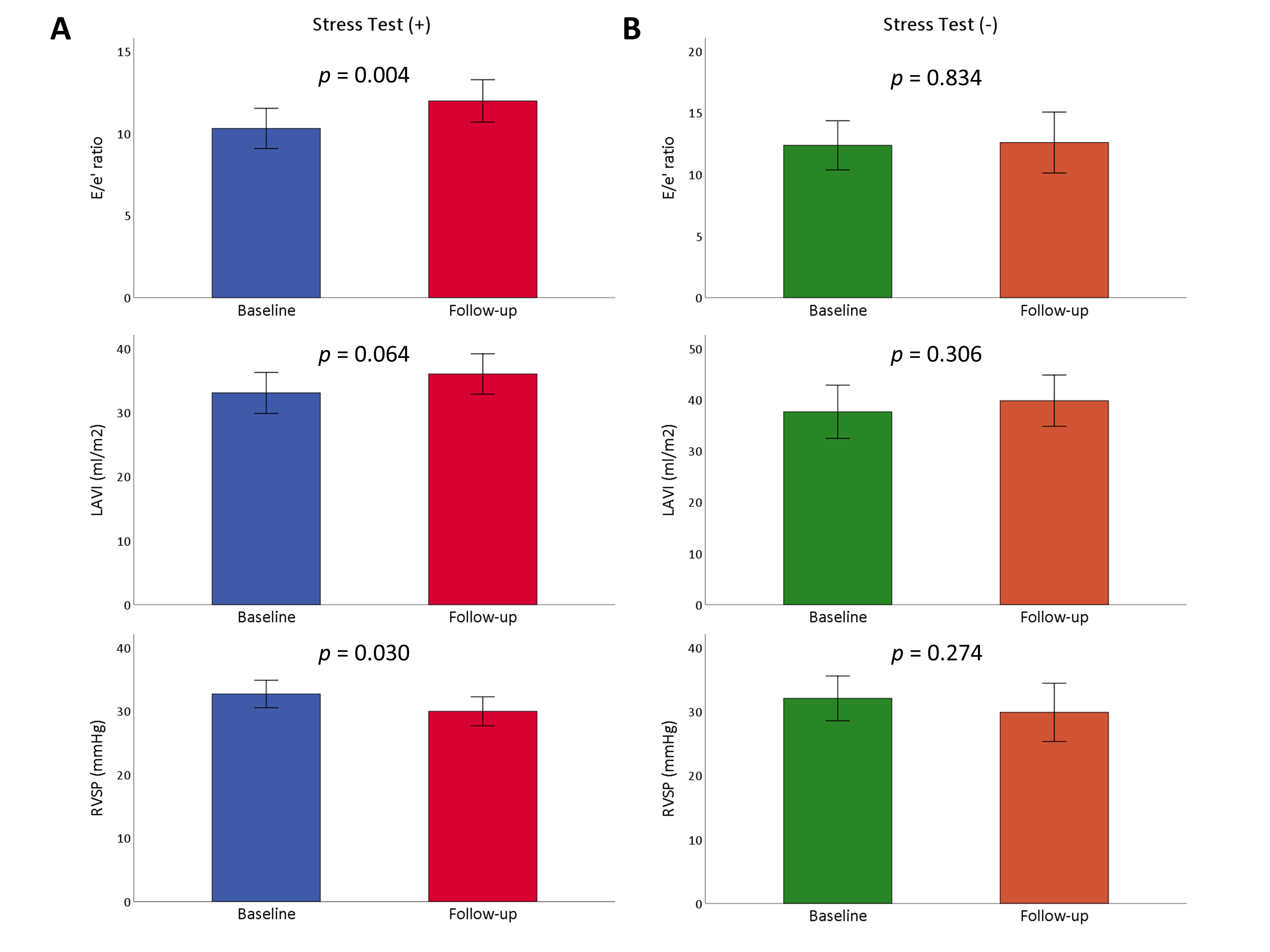

Supplement: Supplementary Figure 4 — Kaplan–Meier curves for cardiovascular death and hospitalization due to heart failure (A) between CAD and no-CAD groups, (B) between stress test (+) and (-) groups. CAD, coronary artery disease; HFpEF, heart failure with preserved ejection fraction. [file Image_4.TIF]

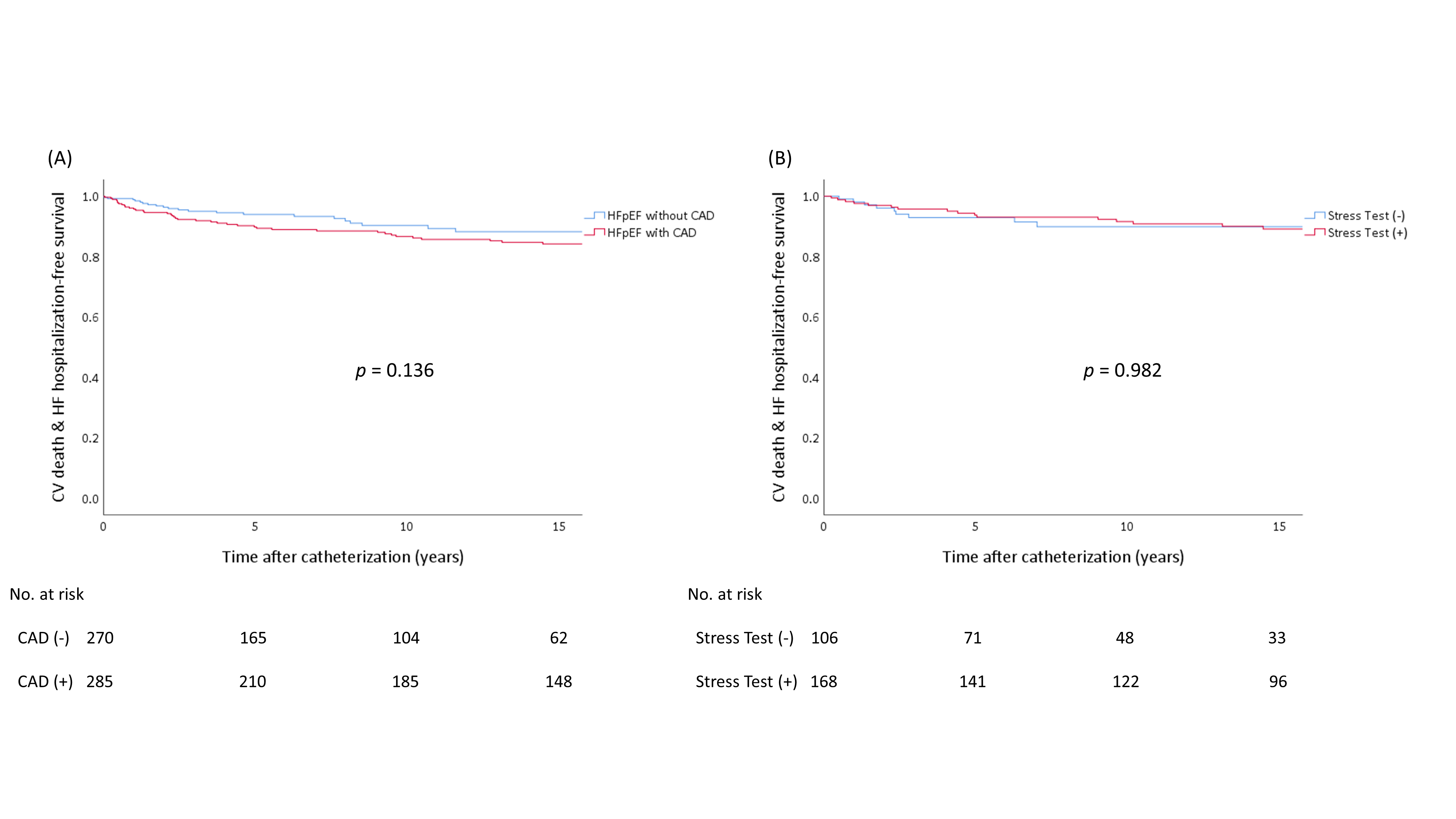

Supplement: Supplementary file 5 [file Image_5.TIF]
